# Prevalence and Risk Factors of MRI Abnormality Which Was Suspected as Sinusitis in Japanese Middle-Aged and Elderly Community Dwellers

**DOI:** 10.1155/2018/4096845

**Published:** 2018-06-12

**Authors:** Saiko Sugiura, Minori Yasue, Yasue Uchida, Masaaki Teranishi, Michihiko Sone, Hirokazu Suzuki, Tsutomu Nakashima, Rei Otsuka, Fujiko Ando, Hiroshi Shimokata

**Affiliations:** ^1^Toyota Josui Mental Clinic, 86-2 Minamidaira, Josui, Toyota, Aichi 470-0343, Japan; ^2^Department of Otorhinolaryngology, National Center for Geriatrics and Gerontology, 7-430 Morioka, Obu, Aichi 474-8511, Japan; ^3^Department of Otorhinolaryngology, Tsushima City Hospital, 3-73 Tachibana, Tsushima, Aichi 496-8537, Japan; ^4^Department of Otorhinolaryngology, Aichi Medical University, 1-1 Yazakokarigane, Nagakute, Aichi 480-1195, Japan; ^5^Department of Otorhinolaryngology, Nagoya University School of Medicine, 65 Tsurumai, Syowa, Nagoya, Aichi 466-8560, Japan; ^6^Ichinomiya Medical Treatment and Habilitation Center, 1679-2 Tomidanagaresuji, Ichinomiya, Aichi 494-0018, Japan; ^7^Section of NILS-LSA, Center for Gerontology and Social Science, National Center for Geriatrics and Gerontology, 7-430 Morioka, Obu, Aichi 474-8511, Japan; ^8^Department of Health and Medical Sciences, Aichi Shukutoku University, 2-9 Katahira, Nagakute, Aichi 480-1197, Japan; ^9^Graduate School of Nutritional Sciences, Nagoya University of Arts and Sciences, 57 Takenoyama, Iwasaki, Nisshin, Aichi 470-0196, Japan

## Abstract

The aims of this study were to determine the prevalence of MRI abnormalities which were suspected as sinusitis in community-dwelling middle-aged and elderly Japanese and to identify risk factors for the MRI abnormality. Brain magnetic resonance imaging (MRI) data from the National Institute for Longevity Sciences, Longitudinal Study of Aging (NILS-LSA) were used for the analysis. Among the 2330 subjects in the NILS-LSA, 1933 participants were categorized as having no MRI abnormality or MRI abnormality using the Lund-Mackay (LM) score. The mean LM score of the participants was 0.88±1.92, and 144 (7.4%) participants had MRI abnormalities which were suspected as sinusitis when it was classified as an LM score greater than or equal to 4. The prevalence of MRI abnormality was significantly higher in participants of older age and the male sex, in participants with obesity, hypertension, bronchial asthma, chronic bronchitis, gout, or hyperuricemia and in ex- or current smokers. A multivariate logistic regression revealed that older age (odds ratio [OR] = 1.17), obesity (OR = 1.54), a smoking habit (OR = 1.71), history of asthma (OR = 3.77), and chronic bronchitis (OR = 2.66) were significant risk factors for MRI abnormality.

## 1. Introduction

Sinusitis is a common disease caused by inflammation of the sinus because of infection or allergy; it is classified as acute or chronic sinusitis based on the duration of the disease. It may cause nasal discharge, nasal obstruction, hyposmia, cough, headache, facial pain, fever, and so on. It is difficult to distinguish sinusitis from rhinitis without an endoscopic examination or imaging examination such as computed tomography (CT). The European Position Paper on Rhinosinusitis and Nasal Polyps (EP3OS) incorporates symptomatic and endoscopic criteria into the clinical diagnosis of rhinosinusitis [1]; sinusitis staging is usually performed with CT. However, the definition of rhinosinusitis is often based only on symptoms in epidemiological studies. Kim et al. reported that the prevalence of chronic rhinosinusitis (CRS) was 10.8% when diagnosed by symptoms only; however, it was 1.2% when diagnosed by symptoms and endoscopy in a Korean population [2].

Various risk factors for sinusitis have been reported previously. Anatomical variations of the sinonasal disease; hazardous environmental and occupational exposures; genetic background; allergic diseases such as allergic rhinitis, asthma, and atopy; immunodeficiency; and smoking were reported as risk factors [1, 3–12]. However, few studies have investigated the associations between sinusitis and medical history in a population-based study.

The aims of this study were to determine the prevalence of MRI abnormalities which were suspected as sinusitis in a community-dwelling middle-aged and elderly Japanese population and to identify risk factors for the MRI abnormality.

## 2. Methods

### 2.1. Participants and Data Collection

The subjects enrolled in this study were those who participated in the 7th wave of the National Institute for Longevity Sciences, Longitudinal Study for Aging (NILS-LSA) from July 2010 to July 2012. The NILS-LSA was a community-based random sample study on aging and age-related diseases that aimed to represent the total middle-aged and elderly Japanese population. The lifestyle of residents in this area is typical of most individuals in Japan. Main purpose of NILS-LSA is systematic observation and description of the process of normal aging in humans. The normal aging process was assessed by detailed examinations including clinical evaluation, sensory functions, body composition and anthropometry, physical functions, nutritional survey, psychological test batteries, and brain magnetic resonance imaging (MRI). Details of the NILS-LSA have been published elsewhere [13] and the protocol and basic data are provided on their webpage [14].

We used data from brain MRI in this study. MRI was performed on a 3-tesla scanner (Toshiba Vischart, Tokyo, Japan). The scanning protocol included a series of axial T1-weighted (TR=500 ms, TE=15 ms) and T2-weighted (TR=4000 ms, TE=120 ms) scans angled parallel to the anterior-posterior commissure line, which included sufficient portion of the paranasal sinus region. MRI findings of mucosal thickening or fluid were considered sinusitis, and MRI findings which were suspected tumor, fungus-like lesion, or other abnormality except for simple sinusitis were excluded from this study. The MRI findings of the nasal and sinuses were staged according to the Lund-Mackay (LM) system [15]. Each paranasal sinus (anterior ethmoid, posterior ethmoid, maxillary, frontal, and sphenoid sinus on the right and left sides) was assigned a score (0 for no opacification, 1 for partial opacification, or 2 for total opacification), and the ostiomeatal complex on each side was also assigned a score (0 for patent, 1 for partially obstructed, or 2 for completely obstructed). Thus, the total score ranges from 0 to 24. An LM score less than 4 was classified as no MRI abnormality, and LM scores greater than or equal to 4 were classified as MRI abnormality which was suspected as sinusitis. An otorhinolaryngologist blinded to the clinical status of the subjects interpreted all MRI series.

Participants were provided with a questionnaire; participants who did not complete the questionnaires about medical history were excluded from this study. The questionnaire included questions about medical history (stroke, hypertension, ischemic heart disease, hyperlipidemia, renal disease, liver disease, diabetes mellitus, peptic ulcer, tuberculosis or pleuritis, bronchial asthma, chronic bronchitis, osteoporosis, rheumatoid arthritis, gout, or hyperuricemia) with possible responses of “none”, “on medication”, “previously on medication”, or “not treated”. “None” was classified as no history of the disease and “on medication”, “previously medicated”, or “not treated” were classified as having a history of the disease. Obesity was defined as a BMI > 25.0 kg/m^2^. A questionnaire about smoking status classified the participants as a “nonsmoker” or “ex- or current smoker”. Alcohol intake was classified as “greater than or equal to 23 g/day” or “less than 23 g/day”. Education level was divided into “more than 9 years” or “less than or equal to 9 years”. Household income was divided into “greater than or equal to 5,500,000 yen/year” or “less than 5,500,000 yen/year”. Physical activity was assessed by the METs score (a multiple of the resting metabolic rate) and obtained through participant interviews with trained interviewers using a semiquantitative assessment method to assess participants' levels of habitual physical activity during leisure time and on the job and their sleeping hours [16]. Physical activity was divided according to whether it was below or above the median of METs × min/year, as follows; “greater than or equal to 700,000 METs × min/year” or “less than 700,000 METs × min/year”.

The study protocol complies with the Declaration of Helsinki and was approved by the Committee on Ethics of Human Research of the National Institute for Longevity Sciences. Written informed consent was obtained from each patient.

### 2.2. Statistical Analysis

Statistical analyses were conducted using the Statistical Analysis System (SAS) version 9.3 (SAS Institute, Cary, NC, USA). Unless otherwise stated, all values are presented as the mean±standard deviation (SD). The chi-squared test for categorical variables and Student's* t* test for continuous variables were used for the univariate analysis. Logistic regression models adjusted for age, sex, and other covariates, which were significantly associated with MRI abnormality in the univariate analysis, were performed to assess risk factors for MRI abnormality. A value of p < 0.05 was considered statistically significant.

## 3. Results

**Figure 1:**
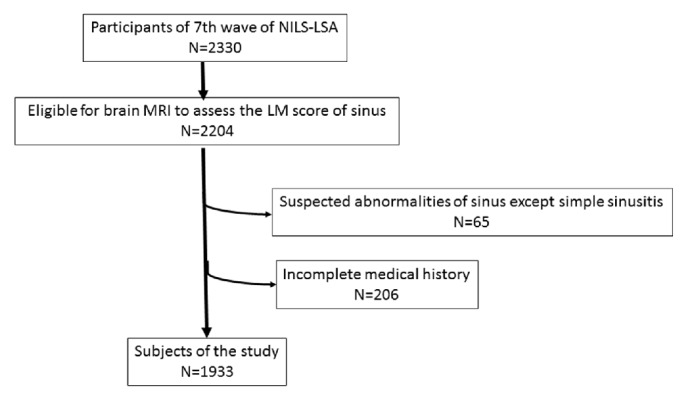
Subjects of the study.

The subjects enrolled in this study were 2,330 adults (1,178 male and 1,152 female) aged 40-91 years. Among the 2,330 participants, 126 had incomplete brain MRI data and 65 were excluded from the study because the MRI findings resulted in a diagnosis of a tumor, fungus-like lesion, or other abnormality except for simple sinusitis. Furthermore, 206 participants who did not complete the questionnaires about medical history were also excluded. Thus, the total number of subjects was 1,933 as described in Figure 1 (967 male, 966 female; age, 60.4±12.4 years).

**Figure 2:**
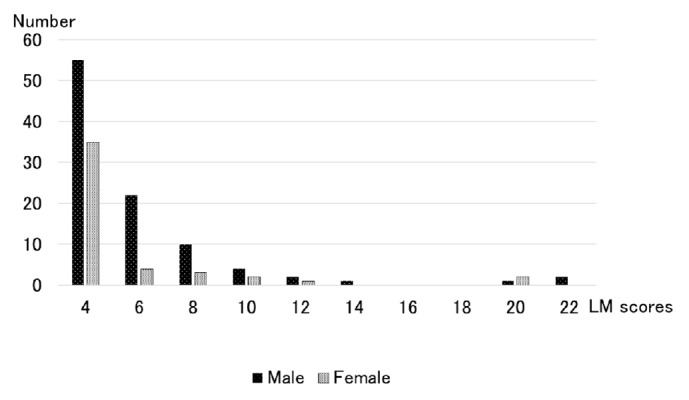
Distributions of Lund-Mackay scores in the MRI abnormality group.

Table 1 shows the mean LM scores according to the sex and age distributions. The mean LM scores of men were greater than those of women in all age groups. The mean LM score peaked in men in their 60s, whereas it peaked in women in their 70s. There were 1,279 subjects with an LM score of 0 points (66.7%) and 410 subjects with an LM score of 1-3 points (21.2%).  Figure 2 shows the distributions of LM scores according to the sinusitis group by sex. The number of subjects became fewer when the LM scores increased to 20 points; there was only one subject with an LM score between 13 and 19 points. However, there were five (0.3%) subjects whose LM scores were greater than 20 points.

Table 2 shows the characteristics of the subjects with and without MRI abnormality which was suspected as sinusitis. Among 1,933 subjects, 144 (7.4%) were suspected as sinusitis. Age; the male sex; obesity; a medical history of hypertension, bronchial asthma, chronic bronchitis, gout, or hyperuricemia; and ex- or current smokers had significantly higher prevalence in the MRI abnormality group.

Table 3 shows the results of the logistic regression analysis. When adjusted for age and sex, a history of hypertension and history of gout or hyperuricemia were not associated significantly with MRI abnormality. The final model adjusted for age; sex; obesity; a history of hypertension, bronchial asthma, chronic bronchitis, gout, or hyperuricemia; and smoking status revealed significant associations between MRI abnormality and older age; obesity; a history of bronchial asthma or chronic bronchitis; and a smoking habit.

## 4. Discussion

In this study, we demonstrated the prevalence of MRI abnormalities which were suspected as sinusitis in Japanese community. The prevalence of sinus abnormalities on MRI ranges from 25 to 85% [3, 17–20]. This range is partially due to variations in study methodology, subjects' characteristics, and definitions of abnormality. Iwabuchi et al. investigated 325 Japanese patients who underwent brain MRI because of suspected cranial nerve disease [17]. An abnormality of the sinuses, which includes not only simple sinusitis but abnormalities such as tumor or fungal infection, was detected in 153 (47.1%) subjects. In our study, 654 (33.8%) subjects had paranasal sinusitis. This result was somewhat less than in Iwabuchi's report; this may due to differences in the characteristics of the subjects and definitions of abnormality.

There were five subjects with severe sinusitis whose LM scores were greater than 20 points. The characteristics of the subjects with severe sinusitis were not different from those of other subjects (data has not shown).

Ashraf et al. reported that the average LM score in patients at ENT clinic with nonsinus symptoms was 4.3, and concluded that an LM score ranging from 0 to 5 may be considered within an incidentally “normal” range [21]. Bhattacharyya reported that an LM score of 2 or 3 was ambiguous for a diagnosis of CRS [22]. Nazri concluded that an LM score exceeding 3 was abnormal [20]. Thus, we analyzed risk factors associated with MRI abnormality as diagnosed by an LM score greater than or equal to 4.

In previous studies, several risk factors of sinusitis have been reported. Obesity is characterized by low-grade systemic inflammation, and Kim et al. reported that the prevalence of obesity was significantly high in the otorhinolaryngologic disease such as chronic otitis media, chronic rhinosinusitis, and chronic tonsillitis [23]. The association between sinusitis and asthma [1, 5, 6, 8, 9, 12] or chronic bronchitis [1, 6, 12] has been clearly established in many studies. Smoking is also established risk factor for CRS [1, 5, 6, 11, 12]. Lee et al. reported that active smoking was significantly associated with CRS in Korean population diagnosed by symptoms only after adjusting for age, sex, residency, household income, education, and occupation in participants aged 40 years and older [11]. Ahn et al. also reported in the same Korean study that significant risk factors for CRS with polyps were the male sex, aging, asthma, a lower level of education, and obesity [5]. Shi et al. surveyed 10,636 Chinese participants and the prevalence of CRS diagnosed by symptoms only was 8.0% [12]. They reported that CRS was prevalent among allergic rhinitis, asthma, chronic obstructive pulmonary disease, and gout. The association between gout and sinusitis was unclear; however, our study also indicated significant associations for both before adjusting for age and sex. Gout is the most prevalent inflammatory arthritis worldwide, although no study has investigated sinus inflammation in gout; thus, further study is desirable. Hypertension had also significantly high prevalence in the sinus group. Dales et al. reported that sinusitis was associated with hypertension in women and discussed about mechanisms linking upper respiratory disorders to hypertension [24].

The male sex and aging were sometimes reported as risk factors for CRS, but in our study, only aging was significant after adjusting for other variables. In the end, significant associations between MRI abnormality which was suspected as sinusitis and older age, obesity, a history of asthma or chronic bronchitis, and a smoking habit remained in the multivariate logistic regression model. The etiology of sinusitis differs by its location. Maxillary sinusitis was reported to be associated with dental illness [25, 26] and fungus infection [27], whereas ethmoid sinusitis or nasal polyps was reported to be associated with allergic rhinitis, atopy, and asthma [6, 8, 9]. A previous population-based study in Malaysia revealed that the most affected sinus was the ethmoid (21.8%) on CT, whereas the most affected sinus on MRI was the maxillary sinus (40.2%) [20]. We reported previously the frequency of sinusitis at each location [28]. The most frequently affected sinus was the maxillary sinus (22.9%), followed by the anterior ethmoid (10.0%) and posterior sinus (9.7%). Both maxillary sinusitis and ethmoid sinusitis were reported to have higher prevalence in male subjects than in female subjects [19]. Further study to analyze the type of sinusitis as well as risk factors and the effects of age and sex will be necessary.

Patients with diabetes mellitus (DM) had significantly worse QOL after endoscopic sinus surgery (ESS) for CRS [29]. Some data indicated an association between gastroesophageal reflux disease (GERD) and upper airway inflammatory disease. Katle et al. showed that there was a causal relationship between GERD and CRS [30]. In our study, there was no association between DM or peptic ulcer and MRI abnormality, although our questionnaire did not include symptoms of GERD.

There are some limitations to this study. First, our questionnaire did not include a question about symptoms of sinusitis such as the existence of nasal discharge and nasal obstruction, and we did not check the endoscopic findings. Thus, our study could not distinguish between acute and chronic sinusitis and between symptomatic and asymptomatic sinusitis or determine the presence of a nasal polyp. Secondly, we assessed sinusitis using brain MRI as substitute for CT. Nazri et al. reported that the sensitivity for detecting abnormalities of the sinuses was higher in MRI than CT, especially for maxillary and ethmoid sinuses [20]. On the other hand, anatomical bone variations cannot be assessed by MRI and are an important risk factor for sinusitis [4, 6]. Despite these limitations, our findings provided valuable results on the prevalence of MRI abnormalities which were suspected as sinusitis in a Japanese community-dwelling middle-aged and elderly population and risk factors for the MRI abnormality.

## 5. Conclusion

Our study showed that the prevalence of MRI abnormalities which were suspected as sinusitis was 7.4% in a Japanese community-dwelling middle-aged and elderly population and that risk factors for MRI abnormality were older age, obesity, a smoking habit, and a history of asthma or chronic bronchitis.

## Figures and Tables

**Table 1:** The LM scores according to the age and sex distributions.

	40-49 years old	50-59 years old	60-69 years old	70-79 years old	80- years old	total
Total(N, average±SD)	488, 0.58±1.44	455, 0.86±1.92	462, 1.10±2.26	401, 1.03±2.10	127, 0.74±1.32	1933, 0.88±1.92
Male(N, average±SD)	238, 0.69±1.61	224, 1.20±2.46	242, 1.41±2.77	206, 1.05±1.64	57, 0.77±1.48	967, 1.07±2.17
Femalel(N, average±SD)	250, 0.48±1.26	231, 0.54±1.10	220, 0.76±1.45	195, 1.01±2.50	70, 0.71±1.19	966, 0.68±1.60

LM scores, Lund-Mackay scores.

**Table 2:** Characteristics of subjects with or without MRI abnormality.

	Total	MRI abnormality	No MRI abnormality	P value^*∗*^
Number	1933	144	1789	
Age (years)^‡^	60.6±12.5	62.7±11.5	60.4±12.6	0.039
Male (N, %)	967 (50.0)	97 (67.4)	870 (48.6)	<.0001
History of stroke (N, %)	63 (3.3)	4 (2.8)	59 (3.3)	1.000^†^
History of hypertension (N, %)	546 (28.3)	52 (36.1)	494 (27.6)	0.029
History of ischemic heart disease (N, %)	66 (3.4)	8 (5.6)	58 (3.2)	0.149^†^
History of hyperlipidemia (N, %)	403 (20.9)	29 (20.1)	374 (20.9)	0.828
History of diabetes mellitus (N, %)	134 (6.9)	10 (6.9)	124 (6.9)	0.995
History of renal disease (N, %)	55 (2.9)	5 (3.5)	50 (2.8)	0.599^†^
History of liver disease (N, %)	66 (3.4)	3 (2.1)	63 (3.5)	0.478^†^
History of peptic ulcer (N, %)	239 (12.4)	17 (11.8)	222 (12.4)	0.832
History of tuberculosis or pleuritis (N, %)	57 (3.0)	4 (2.8)	53 (3.0)	1.000^†^
History of bronchial asthma (N, %)	100 (5.2)	24 (16.7)	76 (4.3)	<.0001
History of chronic bronchitis (N, %)	41 (2.1)	11 (7.6)	30 (1.7)	<.0001^†^
History of osteoporosis (N, %)	142 (7.4)	12 (8.3)	130 (7.3)	0.637
History of rheumatoid arthritis (N, %)	27 (1.4)	3 (2.1)	24 (1.3)	0.448^†^
History of gout or hyperuricemia (N, %)	110 (5.7)	15 (10.4)	95 (5.3)	0.011
alcohol intake ≧23g/day (N, %)	283 (14.6)	25 (17.4)	258 (14.4)	0.337
ex or current-smoker (N, %)	779 (40.3)	84 (58.3)	695 (38.9)	<.0001
education >9 years (N, %)	1655 (85.6)	119 (82.6)	1536 (85.9)	0.290
obesity (N, %)	381 (19.7)	43 (29.9)	338 (18.9)	0.002
physical activity<700,000METs*∗*min/year(N, %)	886 (45.8)	56 (38.9)	830 (46.4)	0.082
household income<5,500,000yen/year(N, %)	889 (46.0)	70 (48.6)	819 (45.8)	0.512

*∗*The Chi-square test for categorical variables and Student's t-test for continuous variables; †Fisher's exact test; ‡average ± SD.

**Table 3:** Logistic regression analysis of risk factors associated with MRI abnormality.

Factors	Crude		Model 1^*∗*^		Model 2^†^	
	Odds ratio (95%CI)	P value	Odds ratio (95%CI)	P value	Odds ratio (95%CI)	P value
Age (every 10 years old)	1.18 (1.03-1.35)	0.020	1.18 (1.03-1.35)	0.019‡	1.17 (1.01-1.37)	0.039
Male	2.18 (1.52-3.13)	<.0001	2.19 (1.52-3.14)	<.0001§	1.51 (0.94-2.40)	0.086
History of hypertension	1.48 (1.04-2.11)	0.030	1.24 (0.85-1.82)	0.272	1.04 (0.70-1.55)	0.842
History of bronchial asthma	4.51 (2.75-7.39)	<.0001	4.74 (2.86-7.84)	<.0001	3.77 (2.17-6.54)	<.0001
History of chronic bronchitis	4.85 (2.38-9.89)	<.0001	4.90 (2.36-10.15)	<.0001	2.66 (1.19-5.98)	0.018
History of gout or hyperuricemia	2.07 (1.17-3.68)	0.013	1.51 (0.84-2.72)	0.168	1.56 (0.85-2.84)	0.151
obesity	1.83 (1.26-2.66)	0.002	1.71 (1.17-2.50)	0.006	1.54 (1.04-2.29)	0.031
ex- or current smoker	2.20 (1.56-3.11)	<.0001	1.68 (1.09-2.59)	0.019	1.71 (1.10-2.66)	0.017

*∗*Adjusted by age and gender. †Adjusted by all factors. ‡Adjusted by gender. §Adjusted by age.

## Data Availability

The data used to support the findings of this study are available from the corresponding author upon request.
